# Structural analysis and optimization of an autonomous robot designed for greenhouse roof cleaning

**DOI:** 10.1038/s41598-025-93687-x

**Published:** 2026-05-27

**Authors:** Ahmed Amin, Xiaochan Wang, Guoxiang Sun, Yinyan Shi, Yongnian Zhang, Qianzhe Cheng, Li Tianhua, Ahmad Noby, Khaled Abdeen Mousa Ali

**Affiliations:** 1 College of Engineering, Nanjing Agricultural University, Nanjing, 210032 China; 2 Agricultural Engineering Research Institute (AEnRI)Agricultural Research Center (ARC), Giza, 12311 Egypt; 3 College of Mechanical and Electronic Engineering, Shandong Agricultural University, Tai’an, 271018 Shandong China; 4 College of Engineering, Al-Azhar University, Cairo, 11884 Egypt; 5 College of Agricultural Engineering, Al-Azhar University, Cairo, 11651 Egypt; 6 College of Engineering, South China Agricultural University, Guangzhou, 510642 China

**Keywords:** CFD, Greenhouse roof cleaner, Robots, Structural analysis, FEA, Mechanical engineering, Engineering, Mathematics and computing

## Abstract

Traditional greenhouse cleaning methods are labor-intensive, prone to human error, and inefficient, often compromising light transmittance and productivity. To address these challenges, this study proposes an autonomous robot designed to clean greenhouse roofs efficiently and reliably. The robot features an integrated cleaning system with adjustable brushes, wipers, and water sprinklers, ensuring optimal performance and significantly improving light transmittance. Powered by a 500 W PV system, it utilizes electric wheels for smooth, stable movement and incorporates a replaceable brush-wiper mechanism for enhancing durability and maintenance efficiency. The design process involved SolidWorks modeling for mass properties, CFD simulations with the k-ε turbulence model to evaluate wind load conditions, and ANSYS structural analysis to confirm durability under extreme wind speeds of up to 126 km/h (ten times greater than normal conditions). Structural tested at different robot’s rotational speeds 25 rpm and 50 rpm confirmed optimal performance at 25 rpm, balancing cleaning efficiency and long-term durability. Additionally, the robot incorporates advanced control unit with sensors for autonomous operation, real-time light transmission monitoring, and navigation capabilities, distinguishing it from traditional manual or semi-automated methods. The results demonstrated robust performance in extreme conditions, surpassing existing systems limited to standard weather. The robot’s performance is limited by speed (0.35 m/s), battery life, roof complexity, maintenance, adaptability, and cost, indicating areas for improvement. Future developments will integrate AI for autonomous decision-making, GPS for precise navigation, and a smart cleaning system to optimize performance based on real-time data, further reducing maintenance costs and ensuring optimal greenhouse lighting.

## Introduction

The rapid growth of the global population has created an urgent need for a 70% increase in food production by 2050, equating to an additional 1 billion tonnes of cereals and 200 million tonnes of meat annually^1^. Achieving this goal requires a significant boost in agricultural productivity, with estimates suggesting an increase of 25–70%, potentially doubling current output levels^2^. However, productivity growth has been sluggish, with critical crop yields rising by only 1% annually which is insufficient to keep pace with population growth^3^. Given the limited availability of arable land, much of the needed production increase must come from intensification rather than expansion^2^. By 2030, the demand for over 100 million hectares of new cropland could result in significant deforestation, exacerbating environmental concerns^3^. This presents a critical challenge: how to sustain global food demands while minimizing environmental impact. The solution lies in advancing agricultural productivity through innovative technologies and sustainable farming practices. Greenhouse technology has emerged as a vital solution to this challenge, enabling year-round cultivation, optimizing resource efficiency, and significantly enhancing crop yields^4,5^. Advanced systems like hydroponics, aquaponics, and precision agriculture are increasingly integrated into greenhouses, maximizing efficiency while minimizing environmental impact^5^. These technologies also allow crop diversification and adaptation to changing climate patterns^6^. The integration of the Internet of Things (IoT) and Wireless Sensor Networks (WSNs) has further revolutionized greenhouse management, enabling precise control and real-time monitoring^7^. Despite challenges such as high initial costs and technical complexity, greenhouse technology holds significant promise for advancing global food security and promoting sustainable agricultural practices^5^.

However, greenhouse technology faces its own set of challenges. One notable issue is the accumulation of dirt on greenhouse roofs, which encourage algae growth, reduce light transparency, and hinder photosynthesis, ultimately affecting crop growth^8^. Particulate matter deposition on greenhouse films can significantly decrease light transmittance, a critical factor for optimal plant development. Various coating agents have been explored to address this, with water-based polydimethylsiloxane showing promise in preventing significant light transmittance loss despite dust accumulation^9^. Additionally, advanced covering materials with adjustable light transmittance have been studied for their effects on leaf photosynthesis, plant physiology, and yield, highlighting the need for interdisciplinary research to enhance energy-efficient cultivation practices^10^. Modern commercial greenhouses typically feature standardized dimensions, with common configurations of 4 m width, 40 m length, and 3 m height, requiring specialized cleaning solutions that can effectively maintain these large surface areas while ensuring structural compatibility.

Manual cleaning of greenhouse roofs poses significant challenges due to the large areas and heights involved. This method not only exposes workers to safety risks, such as falls but also leads to physical strain, including chronic back pain. Additionally, manual cleaning consumes large amounts of water, which is problematic given the current global water crisis. In arid regions, where groundwater often contains heavy elements like iron^11^, manual cleaning can deposit these elements on greenhouse covers, reducing lifespan. Consequently, manual methods are impractical for the extensive areas of agricultural greenhouses, highlighting the need for more efficient cleaning solutions.

Recent research has concentrated on automating greenhouse roof cleaning to address significant challenges associated with manual methods^12,13^. For example, Mijinyawa and Akpenpuun^14^ developed a brush cleaner with a wash mix dispenser, which increased light transmittance by 6–16%. Manor et al.^15^ found that dust can obstruct up to 30% of light energy and explored various cleaning methods using rotating soft brushes. These innovations aim to enhance light transmission and crop productivity by overcoming the limitations of manual cleaning. A comparative analysis of recent greenhouse roof cleaning methods reveals distinct advantages and limitations of each approach, as summarized in Table 1.

**Table 1:** Comparison of roof cleaning methods and performance characteristics.

Cleaning method	Mechanism	Advantages	Disadvantages
Manual cleaning	Hand-operated brushes and water sprayers	- Low initial cost - Adaptable to different surfaces	- Safety risks - Labor intensive - Time-consuming - High water consumption
Brush cleaner with wash mix dispenser (Mijinyawa)^14^	Rotating brushes with cleaning solution	− 6–16% light transmittance improvement - Uniform cleaning	- Limited automation - Fixed cleaning pattern - High maintenance
Sponge wiper robot (Seemuang’s)^16^	Automated sponge wiper blades	− 85–95% dust removal - Low water usage - Gentle on surfaces	- Limited to light dirt - Slow operation speed - Battery dependent
Robot to clean solar panels (Amin et al.)^13^	Rotating brushes without water	− 88–93% light transmittance - Energy autonomous - Real-time monitoring	- Higher initial cost - Weather dependent - Complex maintenance
Proposed system	Integrated solar-powered standard frame with synchronized cleaning mechanisms	- Lightweight design - Wind-resistant design (126 km/h) - Autonomous operation - Optimized energy usage	- Initial setup complexity - Requires technical expertise - Size limitations

Advances in cleaning robots include Seemuang’s^16^ created robotic cleaner using sponge wiper blades, achieving 85–95% dust removal, and Çabuk’s^17^ robot capable of navigating T-shaped iron bars. Amin et al.^18^ demonstrated that improved cleaning by a solar-powered robot increased light transmittance from 88 to 93%, significantly enhancing plant growth in greenhouses. Joint simulation methods using Adams and Matlab have optimized performance, addressing issues such as machine deflection and inconsistent wheel speeds^19^. Structural analysis with software like SAP2000 ensures robust and economical greenhouse construction^20^, while computational techniques, including finite element analysis (FEA), evaluate greenhouse stability under various loads and have revealed discrepancies with European standards^21^.

Structural optimization plays a crucial role in enhancing robot performance while minimizing weight. Techniques such as sensitivity analysis and sequential linear programming optimize robot arms for improved stiffness^22^. FEA compares different arm designs, considering factors like workspace and payload capacity^23^. Topology optimization is valuable in the early design stages, with linear static analyses confirming the safety of optimized arm links^24^. For humanoid robots, integrating structural and controller optimization through multibody system simulation and control co-simulation enhances performance under realistic conditions^25^.

Structural analysis is essential for both industrial manipulators and high-speed parallel robots. It evaluates kinematic and dynamic structures to optimize performance and reliability^26^. While increased model complexity can improve accuracy, it offers diminishing returns beyond a certain point^26^. Lightweight designs can induce vibrations, necessitating careful analysis^27^. FEM is used to predict robot accuracy under various conditions, aiding in the development of efficient and precise robotic systems^28^. Studies like Demir et al.^29^ on mobile robots and Sayyad^30^on self-balancing robots highlight the importance of structural and dynamic analysis.

Integrating solar energy into robotic systems provides significant benefits, especially in remote areas lacking reliable electricity. Solar-powered robots offer a renewable and clean power source. Cruz-Lambert et al.^31^ developed a hybrid solar and battery system for robots, while Santosh Kumar et al.^32^ proposed a solar-powered robot for cleaning photovoltaic panels. Amin et al.^33^ described a dry-cleaning robot for PV panels with a color monitoring system, improving efficiency. Popovski and Ackovska^34^ constructed a solar-powered robotic car using Lego bricks and small solar panels. Amin et al.^18^ created a solar-powered robot to clean greenhouse roofs. These studies underscore the potential of solar energy to enhance robotic systems and promote renewable energy integration.

This research presents an innovative greenhouse roof cleaning robot featuring frame design (4000 mm × 1000 mm), equipped with five 100 W flexible solar panels, brushes, wipers, and water sprinklers. The robot is powered by a 500 W solar power system with strategically placed panels, enabling fully autonomous operation without external power sources. Its lightweight design, with all components weighing no more than 142 kg, and an integrated water tank eliminate the need for external water or power supplies, ensuring uninterrupted operation across the entire greenhouse.

The robot’s cleaning mechanism is highly adaptable, featuring adjustable brush and wiper heights for optimal performance. It utilizes electric wheels for smooth and stable movement over greenhouse roofs, avoiding the complexity of traditional systems. A controllable mechanism allows for the replacement of worn-out brushes and wipers, enhancing durability and maintenance efficiency.

The integrated cleaning system combines brushes, wipers, and water sprinklers in a novel configuration, significantly improving light transmittance for greenhouses. Structural analysis of the brush and wiper components at different speeds (25 rpm and 50 rpm) confirmed optimal performance at 25 rpm, ensuring long-term durability and cleaning efficiency. Additionally, the robot’s structural integrity has been rigorously tested, withstanding extreme wind speeds of up to 126 km/h (ten times greater than normal conditions) surpassing current designs that are typically limited to standard weather conditions.

The robot incorporates a sophisticated control unit with sensors for autonomous operation, real-time light transmission monitoring, and navigation capabilities. These features distinguish it from traditional manual or semi-automated cleaning methods, offering a more efficient and reliable solution for greenhouse maintenance.This paper focuses on the structural analysis and optimization of a greenhouse roof-cleaning robot. The primary objectives of this study are to evaluate the structural integrity of the robot, optimize its design for maximum performance, and enhance its operational efficiency. A detailed structural analysis aims to identify and address potential design flaws, ensure the robot’s durability, and improve its clean greenhouse roofs effectively. The outcomes of this study are expected to offer valuable insights for future research and development in the field of automated greenhouse maintenance, ultimately leading to more sustainable and efficient agricultural practices.

## Materials and methods

This section details the materials, design specifications, and analysis techniques used to develop and optimize the greenhouse roof cleaning robot. The key components and methods are described to ensure reproducibility.

### Materials

The robot is designed to clean the greenhouse roofs from the accumulation of dirt resulting from the accumulation (dust, bird droppings, algae, etc…). This is important to maintain the level of lighting inside the greenhouse, as well as avoid using traditional cleaning methods using labors, which are dangerous to the human factor and also damage the greenhouse cover. It also needs high costs and high-water consumption and has less cleaning efficiency.

**Fig. 1:**
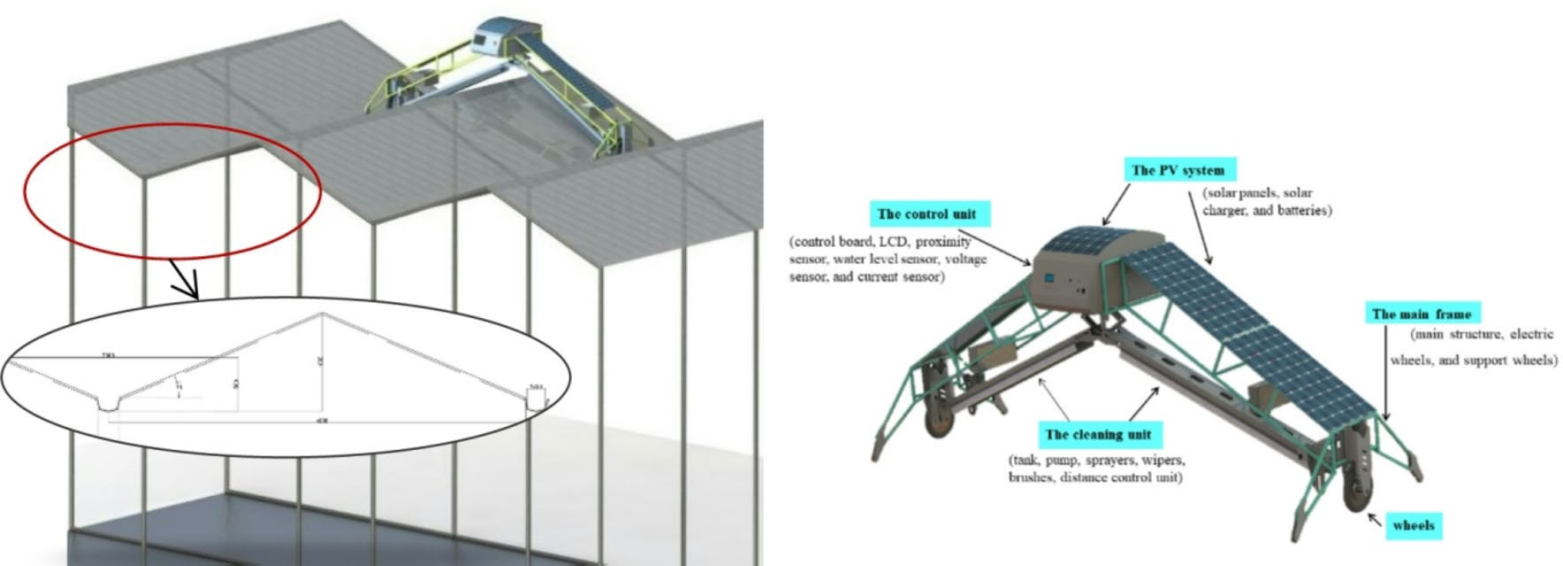
Greenhouse roofs cleaning robot.

The robot, shown in Fig. 1, consists of the following: (1) the main frame (main structure, electric wheels, and support wheels); (2) the cleaning system (tank, pump, sprayers, wipers, brushes, distance control unit); (3) the power supply system (solar panels, solar charger, and batteries, ); and (4) the control unit (control board, LCD, proximity sensor, water level sensor, voltage sensor, and current sensor).

According to Madsen and Jakobsen^35^ considerations made about vehicle principles and options, the concept of the mobile robot was traction, steering, dimensions, frame, motors, and power supply.

**Fig. 2:**
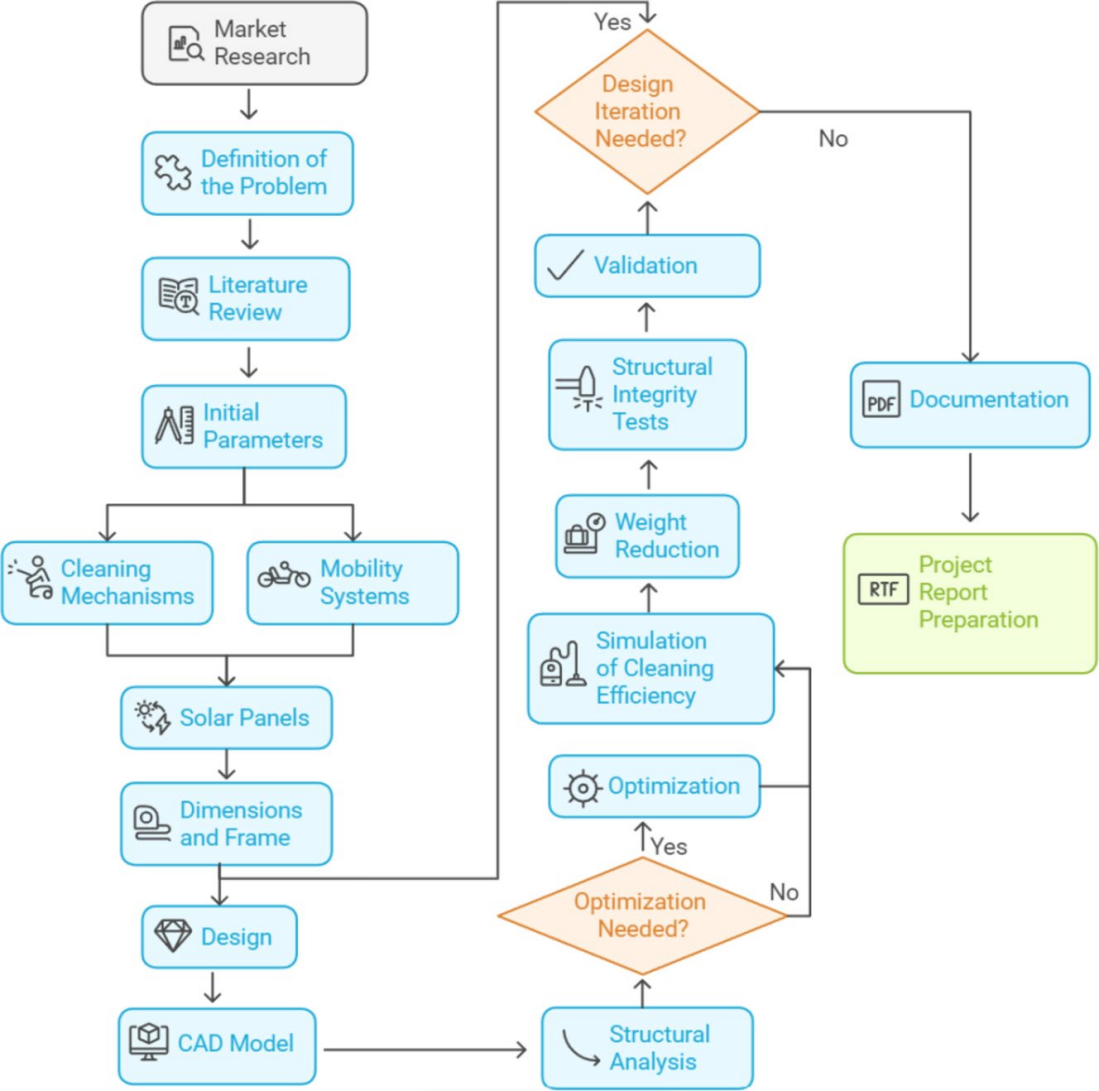
Workflow diagram for the design and development of a greenhouse roof cleaning robot.

The mechanical structure is designed by studying the required working conditions in the field and the desired characteristics of the project using the process steps shown in Fig. 2.

### Main frame

**Fig. 3:**
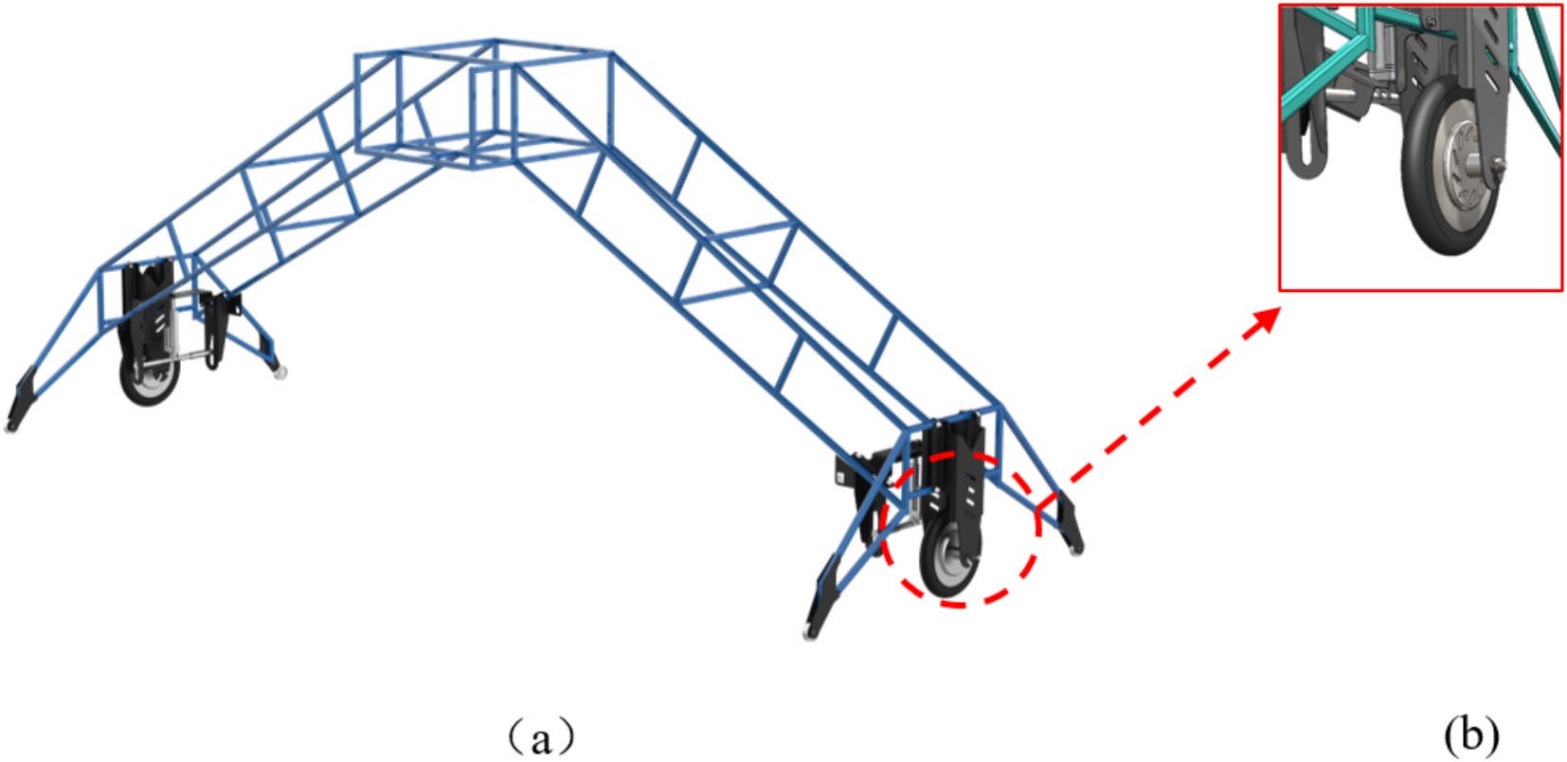
The main frame greenhouse roofs cleaning robot. (**a**) Main structure, (**b**) driver wheels.

The main frame is responsible for carrying all the components of the robot and is also responsible for the movement of the robot on the roof of the greenhouse to perform the task of cleaning the roof of the greenhouse. It consists of two essential parts, as shown in Fig. 3. The first part is the metal structure (carry the robot’s components), and the second is the wheels. The wheels are three wheels on each side; the wheel in the middle is responsible for movement (electric wheel), and the other two wheels are auxiliary.

#### Main structure

The metal structure is made of stainless iron components weighing about 65 kg, in the form of a triangle with a length of each side 2 m and a height of 1 m and the distance between the two sides from the bottom point is 4 m, which is the width of the greenhouse as shown in Fig. 3(a). The robot’s dimensions were specifically designed to accommodate standard greenhouse structures. The main frame width of 4 m aligns with typical greenhouse bay widths. The robot’s standard greenhouse roof height of 3 m and length of 40 m, ensuring efficient coverage of the entire cleaning area. This standardization enables the robot to operate effectively across various greenhouse installations while maintaining optimal cleaning performance. The robot’s compact design, with a height of 1 m and adjustable cleaning width of 0.9 m, allows for complete coverage of the greenhouse roof surface while minimizing structural load. The cleaning system’s configuration ensures uniform pressure distribution and consistent cleaning efficiency across the entire 4 m working width, making it compatible with standard greenhouse panel dimensions.

The robot’s total operational weight includes the main structure (65 kg) and all essential components. The additional weight comprises five 100 W flexible solar panels (2.2 kg each, total 11 kg), four batteries (6-DZF-20.5) weighing 7.5 kg each (total 30 kg), two electric motors (Q-S Motor 12inch 3500 W) at 1.5 kg each (total 3 kg), water tank capacity of 30 L (30 kg when full), cleaning system components including brushes, wipers, and pump (2 kg), and control system components (1 kg). The total operational weight of the robot is approximately 142 kg. This comprehensive weight calculation was incorporated into all structural analyses and simulations to ensure accurate assessment of the robot’s performance under actual operating conditions, particularly for stress distribution, deformation analysis, and wind load calculations.

The analysis considered both static and dynamic loading conditions on standard greenhouse roof structures. Under static conditions, the distributed load of 0.42 kN/m^2^ remains well within the greenhouse roof’s design capacity of 0.96 kN/m². Dynamic load analysis, accounting for the robot’s movement at 0.35 m/s, showed maximum stress concentrations of 0.57 kN/m², including vibration effects. The safety factor of 1.68 confirms the greenhouse structure’s ability to safely support the robot’s operation. Additional finite element analysis of the roof-robot interface demonstrated that the robot’s frame design effectively distributes the load across multiple support points, preventing localized stress concentrations on the greenhouse panels.

#### Driver wheels

Driver wheels of the type (Q-S Motor 12inch 3500 W, 48 V Electric Motorcycle Hub Motor) as shown in Fig. 3(b), which is responsible for the movement of the device and are surrounded by an auxiliary front wheel as well as a rear wheel to increase the balance, and the electrical source of the wheels is the batteries that derive their energy from the solar panels installed on top of the robot.

#### Cleaning system: theory and design

**Fig. 4:**
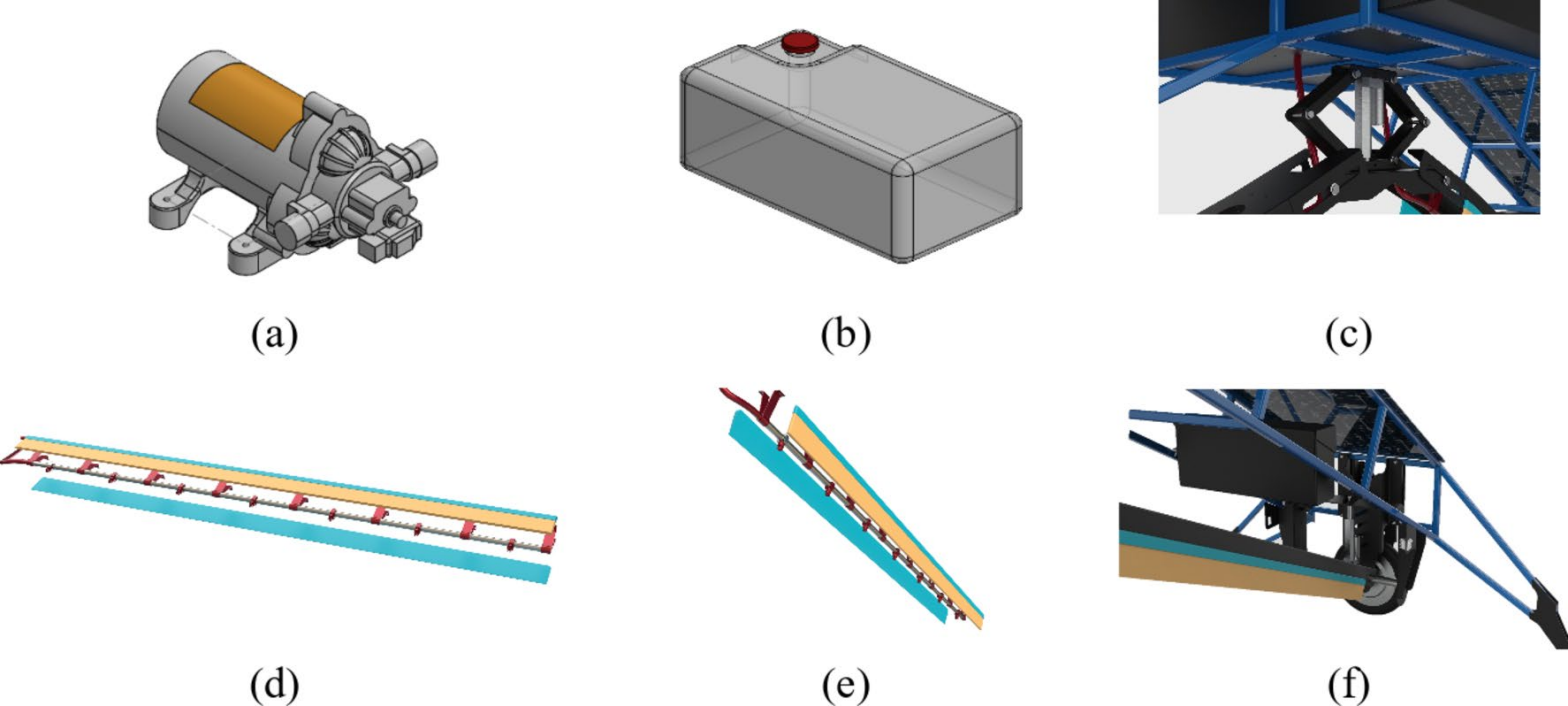
The cleaning system for greenhouse roofs cleaning robot: (**a**) water pump, (**b**) water tank, (**c**) the distance controller, (**d**) water sprayers, (**e**) wipers, (**f**) brushes.

The cleaning system is a critical component of the proposed robot, designed to maintain the cleanliness of greenhouse roofs. This section provides the theoretical background and design principles underlying the system, including the key components and their functional roles. The system integrates mechanical, fluid dynamic, and control principles to ensure efficient and thorough cleaning. The primary components of the cleaning system, as illustrated in Fig. 4, include a 30-liter plastic water tank positioned atop the robot, a water pump (SFDP1-055-060-51) to ensure consistent water flow, sprayers (Teejet Nozzle XR11004VK) for uniform water distribution, wipers (Silicone Rubber [30:40]A) for removing dirt and water, brushes (Nylon 610) for dislodging solid debris, and a distance control motor to maintain consistent contact with the roof surface.

The design and operation of the cleaning system are guided by principles of fluid dynamics, mechanical force application, and control theory. The sprayers are designed to deliver water at a specific flow rate and pressure to ensure uniform coverage. The flow rate Q is determined by the Eq. (1):

1 $$\:\mathrm{Q}=\mathrm{A}\:.\:v$$

where A is the cross-sectional area of the nozzle and *\:v* is the velocity of the water.

The pressure P required to achieve this flow rate is given by the Eq. (2):

2 $$\:P=\frac{1}{2}\:{\uprho\:}{v}^{2}$$

where ρ is the density of water. These equations ensure that the sprayers operate within optimal parameters for effective cleaning. The brushes and wipers apply mechanical forces to remove dirt and debris, with the force F exerted by the brushes being a function of the material properties and the angle of contact. This force is expressed as the Eq. (3):

3 $$\:\mathrm{F}={\upmu\:}\:.\:N$$

where µ is the coefficient of friction between the brush and the roof surface, and N is the normal force.

The 30-degree angle of the brushes and wipers maximizes the tangential force component, enhancing debris removal.

The distance control motor ensures that the brushes and wipers maintain consistent contact with the roof surface. The control algorithm adjusts the motor position based on feedback from sensors, ensuring optimal cleaning performance. The motor’s position x is governed by the Eq. (4):

4 $$x={k_p} \cdot e+{k_d}~.\frac{{\partial \rho }}{{\partial t}}$$

where kp and kd are the proportional and derivative gains, respectively, and e is the error between the desired and actual distance.

The integration of these components and principles ensures a highly efficient cleaning process. The brushes dislodge solid debris, the sprayers apply water to loosen dirt, and the wipers remove the water-dirt mixture. The 100% overlap in water coverage and the precise control of brush and wiper positioning ensure complete and thorough cleaning of the greenhouse roof.

#### Control unit

**Fig. 5:**
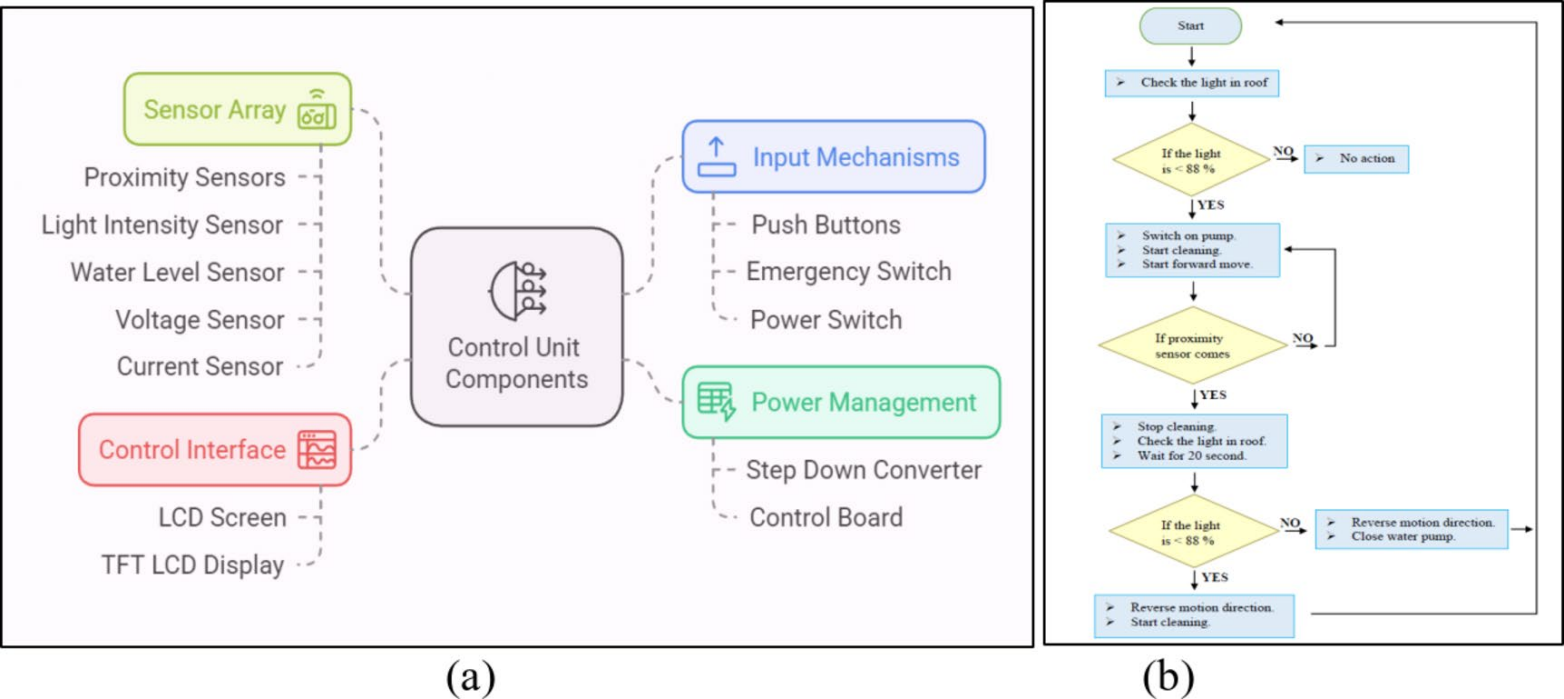
(**a**) control unit components; (**b**) operation flowchart greenhouse roofs cleaning robot.

The control unit acts as the robot’s brain, orchestrating the entire cleaning process with precision. It ensures that each step of the operation follows a well-defined sequence. Table 2 shows the components of the control system, while Fig. 5. presents a flowchart detailing the seamless operation of the robot from the start point of the process to the completion of the cleaning cycle.

The cleaning process start when a sensor detects the light transmission level through the greenhouse roof. If the roof’s cleanliness exceeds 88%, indicating that the roof is adequately clean, the robot remains stationary. However, if the cleanliness falls below 88%, the sensor sends a command to the controller to activate the water pump and start the robot’s forward movement across the roof.

As the robot progresses, it continues to monitor its position until it reaches the designated endpoint. Upon reaching this point, both the water pump and the robot’s movement halt for 20 s. After this pause, the sensor reactivates to reassess the light transmission. If the roof’s cleanliness is still below 88%, the robot reverses its movement while the water pump remains active, returning to the starting point to complete the cleaning cycle. If the light transmission exceeds 88%, the robot reverses without activating the water pump, conserving resources.

**Table 2:** Control unit components.

Components	Description	Quantity
Step down converter	LM2596HVS-ADJ DC-DC Step-Down Module (3 A)	2
Control board	ESP32 room 32	1
LCD screen	TFT LCD Display Module SPI Interface 1.8 Inch 128*160	1
Proximity sensor	Capacitive Proximity Sensor	2
Light intensity sensor	BH − 1750	1
Water level sensor	KIT0139	2
Voltage sensor	DC0-25 V	1
Current sensor	ACS712–30 A	1
Push buttons	Metal Momentary Push Button 2 pin 16 mm	3
Emergency switch	A16-11ZR Emergency Stop Switch 16 mm	1
Power switch	60 A	2

#### Power source (PV) system

Figure 1 illustrates the components (solar panels, solar charger, and batteries) of the energy system responsible for efficiently powering the robot during the cleaning process.

Solar panels are the key elements responsible for converting sunlight into electrical energy. The system consists of five flexible panels, each with a capacity of 100 watts (model SWF-100 W), providing a combined total of 500 watts. The panels are strategically arranged to maximize solar exposure: two panels are mounted on each side of the robot, and the fifth panel is positioned on top. This flexible panel design ensures that the robot can efficiently capture sunlight from various angles throughout the day, supporting continuous and uninterrupted operation, as shown in Fig. 1.

The charge controller is of type MPPT (SSD600W), and it is responsible for effectively regulating and distributing the electrical charges generated by the solar panels. The charge controller operates using Maximum Power Point Tracking (MPPT) technology, which enables optimal utilization of the solar panels under various lighting conditions. It ensures that energy flows efficiently into the batteries without significant losses, thereby enhancing battery life and increasing the overall efficiency of the system.

The batteries serve as the energy storage for the robot. The system consists of four batteries of type (6-DZF-20.5 (12 V, 20.5Ah)), designed to store the electrical energy generated by the solar panels during the day for later use, even during periods of low sunlight or nighttime. These batteries are characterized by their high capacity to retain charge for extended periods, ensuring that the robot operates efficiently throughout the required working time.

### Methods

To evaluate the robot’s performance under operational conditions, performance-based simulations were conducted using Computational Fluid Dynamics (CFD) and Finite Element Analysis (FEA). CFD simulations were performed to analyze the robot’s aerodynamic stability under wind loads. The k−ϵ turbulence model was used, under extreme wind speeds of up to 126 km/h (ten times greater than normal conditions) applied at the inlet and atmospheric pressure at the outlet. A structured mesh, refined near the robot’s surface, captured boundary layer effects. The results, including pressure distributions and drag forces, were used to optimize the robot’s design for improved aerodynamic efficiency. Additionally, FEA was conducted to assess the structural integrity of critical components, such as brushes and wipers. The 3D CAD model was imported into the FEA software, with material properties assigned and operational loads applied. A fine mesh, refined at stress points, ensured accurate results. Stress and strain distributions were analyzed to identify potential failure areas, guiding design improvements for enhanced durability.

#### Mesh

**Fig. 6:**
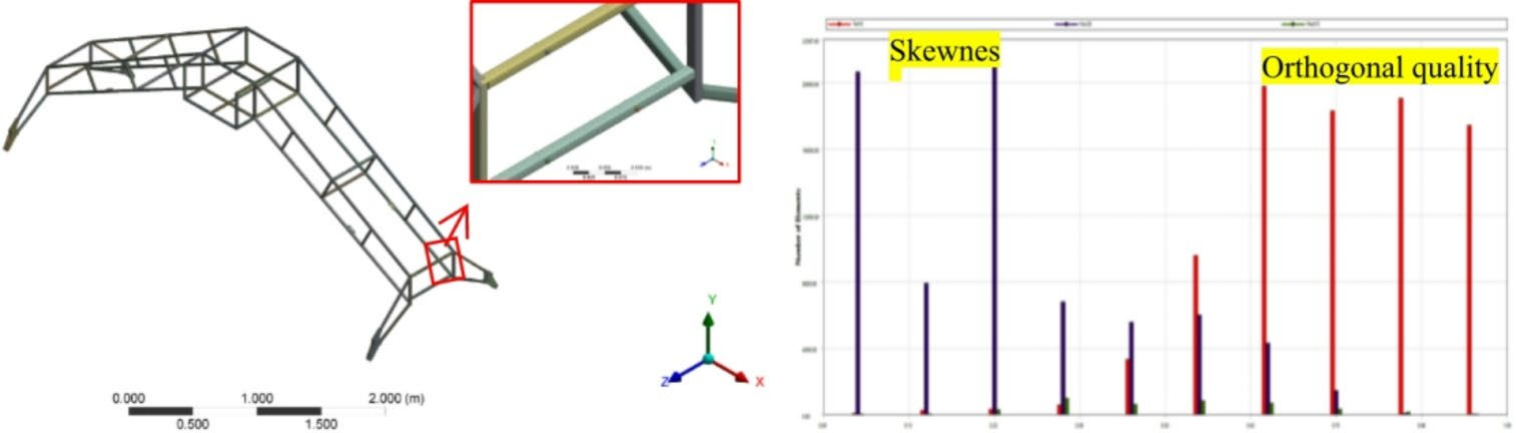
Mesh of the robot structure model along with the spectrum of skewness and orthogonal quality mesh metrics.

A static structural analysis and finite element analysis (FEA) were conducted on a three-dimensional model that accurately represented the robot’s actual dimensions. The default mesh was refined by incorporating additional controls to enhance mesh quality, with a particular focus on element distribution, skewness, and orthogonal quality. Various meshing techniques, including proximity and curvature functions, were employed to ensure a mesh quality score of at least 0.8. Figure 6 illustrates the mesh metrics spectrum, highlighting standard skewness and orthogonal quality values^36^.

The robot’s geometry features a length of 4000 mm and a height of 1000 mm, with a mesh size of 1 mm × 1 mm. The meshing method achieved an average element quality of 0.81, an average skewness of 0.22, and an average orthogonal quality of 0.85, demonstrating the high mesh quality employed in this analysis. These findings are consistent with the results reported by Fatchurrohman and Chia^37^.

#### Finite method and CFD implementation

**Fig. 7:**
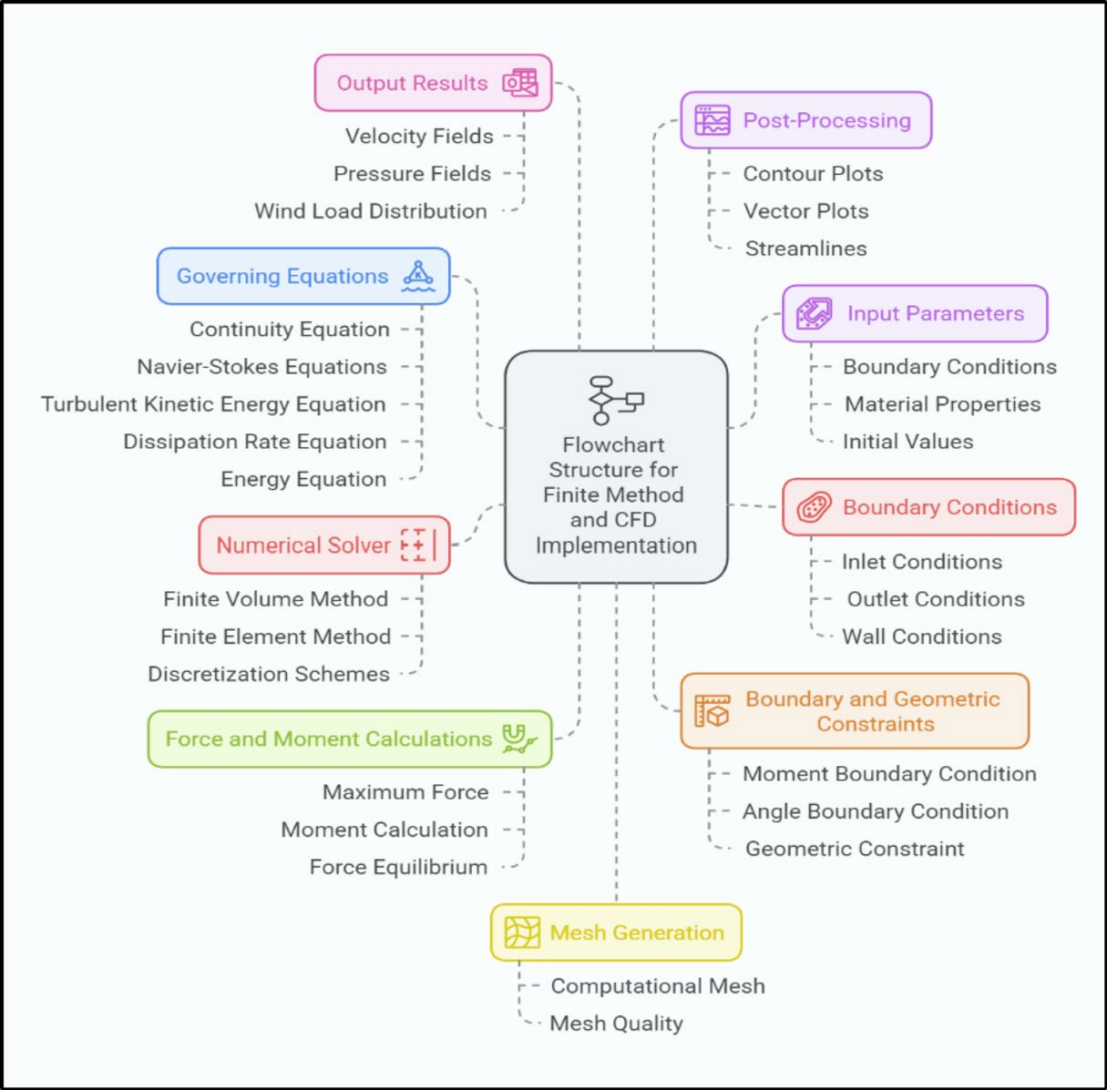
Flowchart structure for finite method and CFD implementation.

The flowchart in Fig. 7 presented outlines the step-by-step implementation of the finite method and computational fluid dynamics (CFD) framework used in this study. The process begins with defining input parameters, such as boundary conditions, material properties, and initial values, followed by mesh generation to discretize the computational domain. The governing equations including continuity, Navier-Stokes, turbulent kinetic energy (*k*), dissipation rate (∈), and energy equations are then applied to model the fluid flow and thermal behavior. Boundary conditions, such as wind velocity and pressure, are incorporated to simulate real-world scenarios. The numerical solver iteratively computes the solution, ensuring convergence and accuracy. Wind load calculations, force and moment analyses, and geometric constraints are integrated to evaluate structural responses.

In Simcenter STAR-CCM+, selecting the right solver settings is essential for accurate CFD simulations. The solver type (steady-state or transient) depends on flow conditions, and convergence criteria focus on reducing residuals, maintaining low flux imbalances, and stabilizing key quantities. Numerical schemes like high-resolution balance accuracy and stability, while turbulence models such as k-ε capture turbulent effects efficiently. Properly configured settings ensure reliable and robust CFD results, meeting the required accuracy and stability standards.

The k-ε (k-epsilon) turbulence model is widely used in Computational Fluid Dynamics (CFD) for simulating turbulent flows. It is a two-equation model that provides a robust and efficient approach to modeling turbulence by solving two transport equations: one for the turbulent kinetic energy ($\:\mathrm{k}$) and one for the rate of dissipation of turbulent kinetic energy (*∈*). The model is popular due to its simplicity, computational efficiency, and reasonable accuracy across a wide range of engineering applications^38^.

The transport equations for the k-ε turbulence model are given by:

Turbulent Kinetic Energy ($\:\mathrm{k}$):

5 $$\:\frac{\partial\:k}{\partial\:t}+{U}_{j}\frac{\partial\:\mathrm{k}}{\partial\:{x}_{j}}=\frac{\partial\:}{\partial\:{x}_{j}}\:\left[\left(v+\frac{{v}_{t}}{{\sigma\:}_{k}}\right)\frac{\partial\:\mathrm{k}}{\partial\:{x}_{j}}\right]+{P}_{k}\:-\in$$

Rate of Dissipation (*∈*):

6 $$\:\frac{\partial\:\in}{\partial\:t}+{U}_{j}\frac{\partial\:\in}{\partial\:{x}_{j}}=\frac{\partial\:}{\partial\:{x}_{j}}\:\left[\left(v+\frac{{v}_{t}}{{\sigma\:}_{\in}}\right)\frac{\partial\:\in}{\partial\:{x}_{j}}\right]+{{C}_{1\in}\frac{\in}{k}P}_{k}\:-{C}_{2\in}$$

Where:

- $\:{U}_{j}$ = velocity component in the j -direction
- ( *\:v*) = kinematic viscosity
- ($$\:{v}_{t}$$) = turbulent viscosity
- ($\:{P}_{k}$) = production of turbulent kinetic energy
- ($\:{\sigma\:}_{k}$) and ($\:{\sigma\:}_{\in}$) = model constants
- ($\:{C}_{1\in}$) and ($\:{C}_{2\in}$) = empirical constants

The k-ε model’s effectiveness lies in its balance between computational efficiency and accuracy, making it well-suited for a variety of engineering problems involving turbulent flow.

##### Continuity equation

7 $$\:\frac{\partial\:{\uprho\:}}{\partial\:t}+\nabla\:.\left({\uprho\:}\mathrm{u}\right)=0$$

Where: *ρ* is the fluid density, u is the velocity vector, t is time.

This equation ensures that mass is conserved in the fluid flow.

##### Navier-Stokes equations

8 $$\:\rho\:\left(\frac{\partial\:\mathrm{u}}{\partial\:t}+\mathrm{u}.\:\nabla\:\:\mathrm{u}\right)=-\:\nabla\:\rho\:+\mu\:{\nabla\:}^{2}u+f$$

Where: p is the pressure, µ is the dynamic viscosity of the fluid, *f* represents external forces.

These Eqs. (7) and (8) describe the motion of the fluid and are used to calculate the velocity and pressure fields.

##### Energy equation

9 $$\:\rho\:{\complement\:}_{p}\left(\frac{\partial\:\mathrm{T}}{\partial\:t}+\mathrm{u}.\:\nabla\:\:\mathrm{T}\right)=-\:\nabla\:.\:\left(k\nabla\:T\right)+{\Phi\:}$$

Where: T is the temperature, cp. is the specific heat capacity, *k* is the thermal conductivity, Φ represents viscous dissipation.

This equation is used if thermal effects are considered in the simulation.

##### Wind load assumption

The wind load acting on the greenhouse cleaning system was calculated based on the wind pressure distribution over the surface of the system. The wind pressure (P_w_) was determined using the following equation:

10 $$\:{P}_{w}=\frac{1}{2}\:{\uprho\:}{v}^{2}{C}_{P}$$

Where: $\:{\uprho\:}$ is the air density (typically 1.225 kg/m^3^at sea level), *\:v* is the wind velocity,

$\:{C}_{P}$, is the pressure coefficient, which depends on the shape and orientation of the structure.

The wind velocity (*\:v*) was assumed to be 126 km/h, which is a typical value for moderate wind conditions in Nanjing city, China.

##### Integration into CFD simulations

The wind load was incorporated into the CFD simulations as a boundary condition. The wind velocity (v) was applied as an inlet boundary condition, and the pressure distribution (*P*_*w*_) was calculated using the Navier-Stokes Eq. (8).

The pressure coefficient (C_p_) was derived from the CFD simulations by analyzing the pressure distribution on the roof of the greenhouse cleaning system.

#### Brush and wiper design

The main body is designed as a rectangular brush that covers the cleaned material. It is installed at an angle that allows for dust removal before spraying cleaning material (water or Cement Removal Agent (CRA)) and then comes the wiper to remove the dirty cleaning water. The brush and wiper, combined with the water spray (Nozzles), effectively clean most of the dirt from all greenhouse roofs. In the cleaning process, dirty water flows down by the wiper. The cleaning capacity of a cleaning machine refers to the maximum area that can be cleaned in a given unit of time while maintaining cleaning efficiency. This value is calculated by multiplying the cleaning width by the maximum travel speed. Equation (7)^39^.

11 $$\:{N}_{\mathrm{m}\mathrm{a}\mathrm{x}}=\:B\:\times\:\:{V}_{\mathrm{m}\mathrm{a}\mathrm{x}}$$

where: N_max_ represents the maximum cleaning capacity; B stands for the cleaning width in meters; V_max_ is the maximum cleaning speed in meters per second.

The formula above shows that the cleaning capacity of the robot is directly proportional to its cleaning width and speed. When the cleaning robot reaches its maximum cleaning capacity, the cleaning width should be maximized, and the driving speed should be increased. However, excessively fast cleaning width and driving speed can negatively impact the cleaning effectiveness of the robot, potentially leading to incomplete or subpar cleaning. Therefore, it’s important to set the cleaning width and working speed at reasonable levels to enhance the robot’s cleaning performance. Based on calculations and experimental analysis, the suitable brush width is W = 0.4 cm, and the cleaning width is 0.9 cm. To achieve the desired cleaning effect, it is determined that the light transmittance of the cleaning robot should exceed 88%. This requires a cleaning time of over 30 min for one line, meaning the cleaning robot’s driving speed should not exceed 0.35 m/s.

**Fig. 8:**
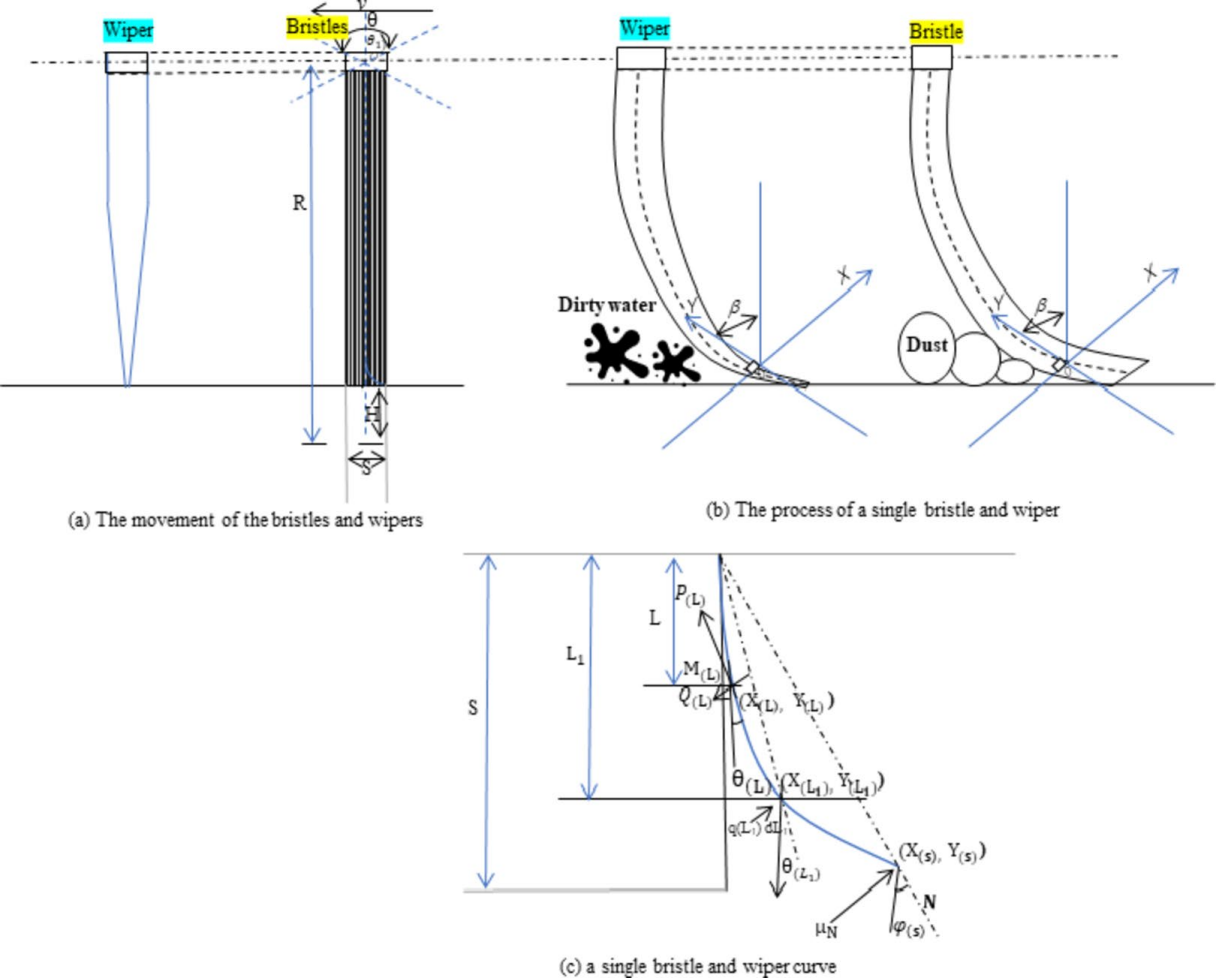
The brush and wiper state diagram.

The filaments of the brush and wiper are modeled using elastic deformation theory, and the state diagram of the bristles and wipers during movement can be seen in Fig. 8a. The robot speed does not exceed 0.35 m/s, and the angle between gravity, makes the effect of air resistance, gravity, and force on the filaments negligible. The bristles and wipers are flexible and conform to the theory of elastic deformation. This theory suggests that the tips of the bristles and wiper are the points of contact, with the individual bristles sweeping the dust and the wipers removing the water, as demonstrated in Fig. 8b. A Cartesian coordinate system is constructed on the XOY plane, with one end of the bristles and wipers as the origin, and the Y-axis representing the filaments along the vertical direction. S represents the length of the bristles and wipers, and the condition of the bristles and wipers is illustrated in Fig. 8c.

## System design and functionality

**Fig. 9:**
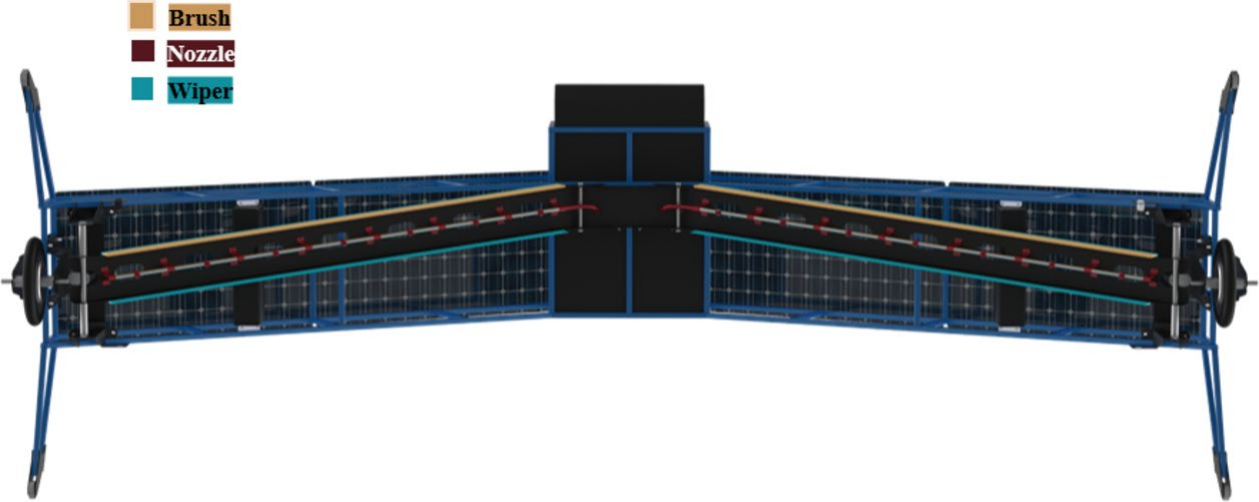
Brushes and wipers at a 30-degree angle to the robot’s axis.

The brushes and wipers are positioned at a 30-degree angle to the robot’s axis, optimizing the removal of dirty water from the lower sections of the greenhouse roof. The wipers are activated to remove the dirty cleaning water as shown in Fig. 9, working in conjunction with the water spray to efficiently remove dirt. During the cleaning process, the dirty water is directed off the roof by the wipers, ensuring thorough cleaning. The system’s cleaning capacity is determined by the maximum area that can be cleaned within a specific time frame while maintaining high cleaning efficiency.

## Mathematical modeling

### Bending moment and shear force

The bending moment M of the bristles and wipers is given by:

12 $$\:M=-EI\frac{\partial\:{\uptheta\:}}{\partial\:l}$$

where: M Bending moment; EI is the flexural stiffness of the bristles or wipers; $\:{\uptheta\:}$ Angle of deflection, *\:l* is the length of the bristles or wipers.

### Shear equilibrium equation

In the bent state of the bristles (Fig. 8c), the shear equilibrium equation is:

13 $$\:Q-N\:sin\left[{\uptheta\:}\left(L\right)-\:\phi\:\left(S\right)\right]-\mu\:N\:cos\left[{\uptheta\:}\left(L\right)-\:\phi\:\left(S\right)\right]=0$$

Q: Shear force, N: Normal force, µ: Friction coefficient, ϕ(S): Angle of the surface at point S.

### Boundary and geometric conditions

*Boundary condition*: At the tip of the bristles (wipers), the bending moment is zero.

14 $$\:M\left(S\right)=0$$

Geometric condition: At the origin of the bristles (tangent to the Y-axis):

15 $$\:\theta\:\left(0\right)=0$$

*Contact condition*: The coordinates of the bristle (wipers) tip in contact with the surface are (x(s), y(s)), with the distance from the origin.

16 $$\:\sqrt{{x}^{2}\left(S\right)+{y}^{2}\left(S\right)}=R$$

### Model discretization

The angle θ between the bristles/wipers and the coordinate axis is determined using model discretization. The function θ(L) is approximated as:^26^:

17 $$\:{\theta\:}_{\left(L\right)}={\sum\:}_{i-0}^{n}{C}_{i}{L}^{i}$$

where C_i_ are coefficients to be determined, and *n* = 6.

Based on the calculations provided above, the brush and wiper parameters are as Table 3.

**Table 3:** Brush and wiper parameters.

Brush parameters	Wiper parameters
*Bristle length*: 70 mm	*Base width*: 8.00 mm
*Bristle diameter*: 0.5 mm	*Tip width*: 1.00 mm
*Bristle density*: 1050 kg/m³	*Total height*: 70 mm
*Friction coefficient*: 0.25	*Material*: 30:40 A silicone rubber
*Elastic modulus*: 8.3 × 109 Pa8.3 × 109 Pa *Total brush head length*: 2000 mm	*Elastic modulus*: 1.5 × 106 Pa1.5 × 106 Pa

These parameters, along with Eqs. (12), (13), (14), (15), (16), (17), model the elastic deformation of the bristles and wipers. The bending moment M and shear force Q are derived from flexural stiffness EI and geometric conditions, ensuring normal force generation at the bristle and wiper tips. The model’s discretization determines the angle θ, enabling efficient cleaning through coordinated brush, wiper, and water spray action.

#### The limitations of robot performance and utilization features

##### Limitations of robot performance

- Operational speed and efficiency: The robot’s operational speed is currently limited to 0.35 m/s to ensure stability and cleaning effectiveness. This may result in longer cleaning times for large greenhouses.
- Battery life and energy consumption: The robot’s battery life is limited by its energy consumption, which is influenced by factors such as motor power, water pump usage, and operational time.
- Cleaning effectiveness on complex surfaces: The robot’s cleaning effectiveness may be reduced on surfaces with complex geometries or heavy contamination.
- Durability and maintenance: The robot’s components, such as brushes and wipers, are subject to wear and tear over time, requiring periodic maintenance and replacement.

##### Limitations of utilization features

- Adaptability to different greenhouse designs: The robot’s design is optimized for Venlo-type greenhouses, and its performance may vary in greenhouses with different roof designs or materials.
- Cost and accessibility: The initial cost of the robot may be a barrier for small-scale greenhouse operators.

## Results and discussion

### Structural analysis

**Fig. 10:**
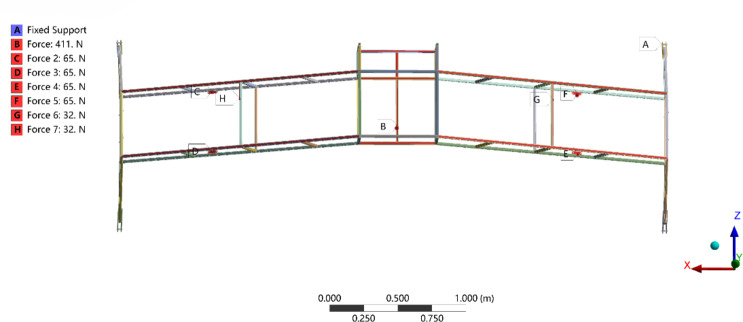
Load applied on the main structure.

The material properties used in the finite element analyses were obtained from the manufacturer, including a Young’s Modulus of 210 GPa, Poisson’s Ratio of 0.3, Density of 7.85 kg/mm³, and both Tensile Yield and Compressive Strengths of 235 MPa. The analysis incorporated the total operational weight of 142 kg, including all components and attachments, to ensure realistic load conditions. The simulation model accounted for the distributed weight of solar panels, batteries, motors, full water tank, and all auxiliary systems, providing a comprehensive assessment of the robot’s structural integrity under actual operating conditions. Details of the structure and the applied forces are shown in Fig. 10.

**Fig. 11:**
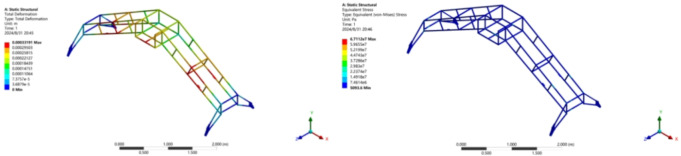
Total stress and deformation of the structure.

Figure 11 illustrates the simulation results of the system. The deformation in the frame is primarily concentrated in the areas where the batteries are mounted on the sides and where the water tank is installed on the top. However, these deformations do not compromise the overall performance of the structure. In terms of stress distribution, stress accumulation was observed at the junction between the upper and side frames, though it remains within acceptable limits and does not induce turbulence.

### Analysis of the cleaning modules

The cleaning unit consists of two brushes, two wipers, and water sprinklers that cover the greenhouse roof surface designed to clean it. Given that the unit operates under load conditions, it is essential to consider deformation and stress under static loading when designing the structural components. The wipers play a vital role in removing dirty water and enhancing the overall cleaning efficiency of the brushes. To ensure a robust and consistent design, finite element simulations were performed on the brushes, wipers, and bristles. Detailed conditional and static structural analyses of these components are presented in this section.

#### Brush and wiper

**Fig. 12:**
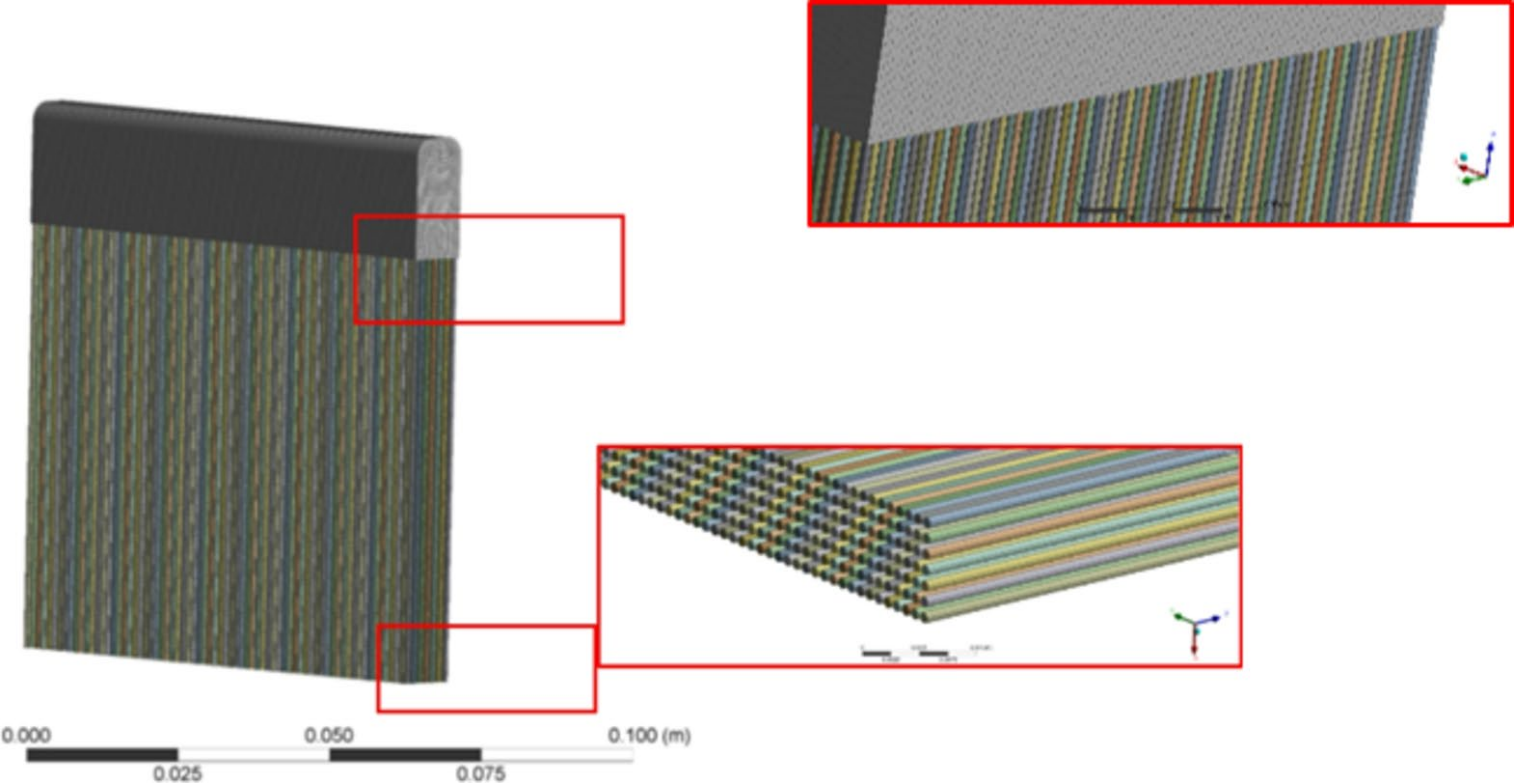
Mesh model of the brush.

The brush is pulled by a motor (Q-S Motor, 12-inch, 350 W, 48 V) without rotation. Modal analysis was conducted on the brush model to determine the corresponding mode shapes under static load conditions. The brush model was imported into ANSYS Workbench and meshed using tetrahedral elements with a body size of 1 mm, as shown in Fig. 12. The number of elements 200,893 nodes. The meshing method used in this study demonstrated high quality, achieving an average element quality of 0.85, an average skewness of 0.32, and an average orthogonal quality of 0.85. These metrics confirm the effectiveness and reliability of the meshing approach employed.

**Fig. 13:**
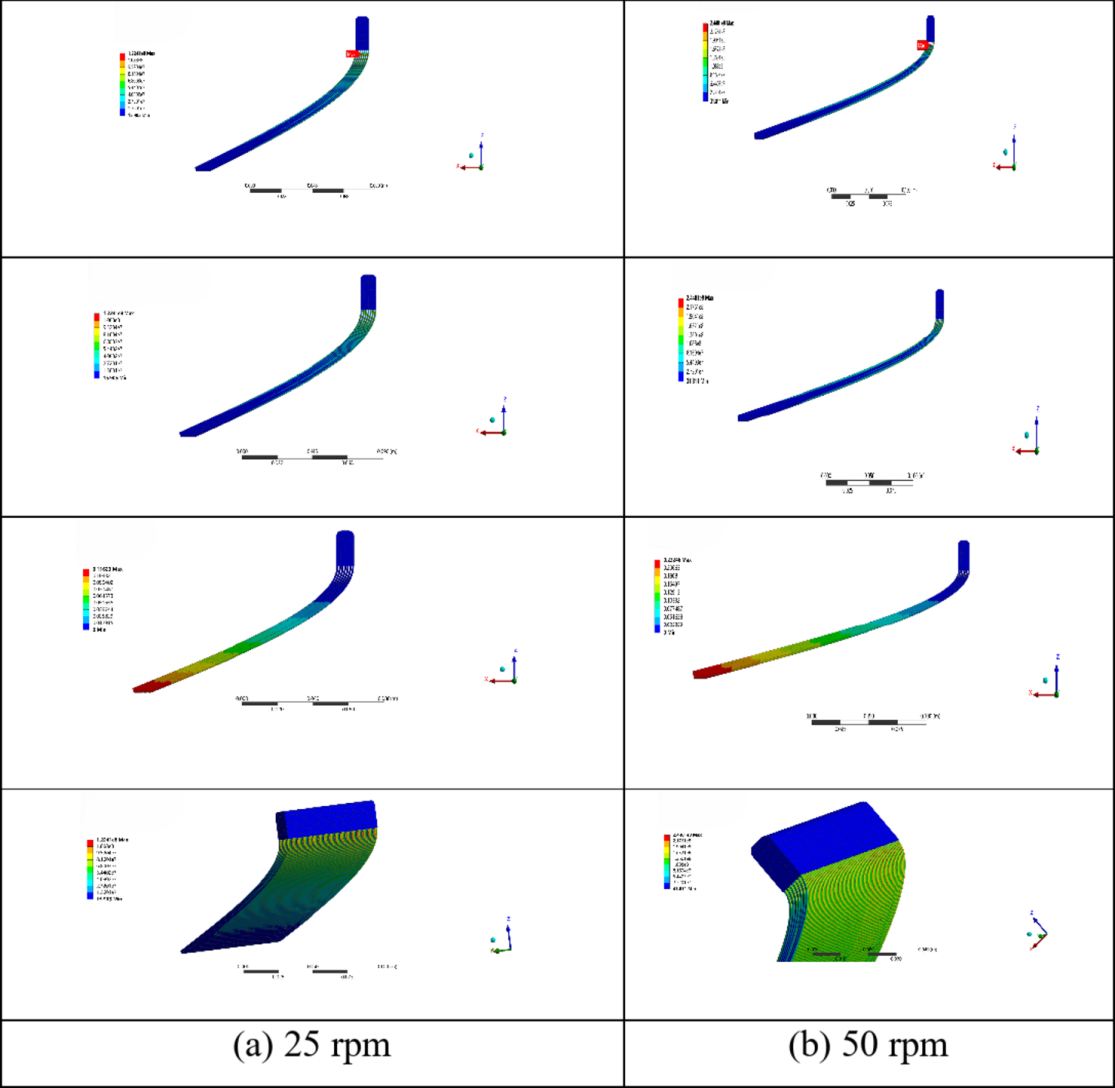
The brush model shapes (**a**) motors speed 25 rpm (**b**) motors speed 50 rpm.

The simulation results in Fig. 13(a), (b) compare the structural behavior of the brush at 25 rpm and 50 rpm, highlighting the impact of rotational speed on stress distribution and deformation. This part of the study specifically examines rectangular filament rows that are 9 mm wide and 2000 mm long, mounted on rectangular panels connected to the cleaning robot. As the brush pulls with the bristles in contact with the greenhouse surface, the bristles experience complex loading at their edges, distributed along all the bristles and panels. Each bristle, modeled as a long cantilever beam, undergoes tangential deflection to effectively sweep dust particles. Using ANSYS Workbench, the bristles were meshed with tetrahedral elements sized at 0.5 mm, and a combined loading scenario incorporating pulling forces from the drive motor and resistance forces from the greenhouse roof was applied to the bristle tips to evaluate deformation and stress patterns. The static analysis results reveal that with small deflections, bristle deformation is 2 mm, while with large deflections, it reaches 42 mm, demonstrating the significant impact of load conditions. At both speeds, stress concentrates at the base of the arm, with higher levels at 50 rpm due to increased loading, indicating the need for stronger materials and fatigue resistance at higher speeds. Deformation is more pronounced at 50 rpm, which could affect cleaning efficiency and operational accuracy, whereas 25 rpm provides more controlled deformation, enhancing stability. Although 50 rpm offers faster operation, it induces higher stress and deformation that could lead to wear over time, suggesting that 25 rpm offers a balanced approach for long-term durability. To optimize performance, reinforcing critical stress points and selecting materials with higher fatigue resistance could mitigate the observed adverse effects, particularly at higher speeds.

#### Wiper

**Fig. 14:**
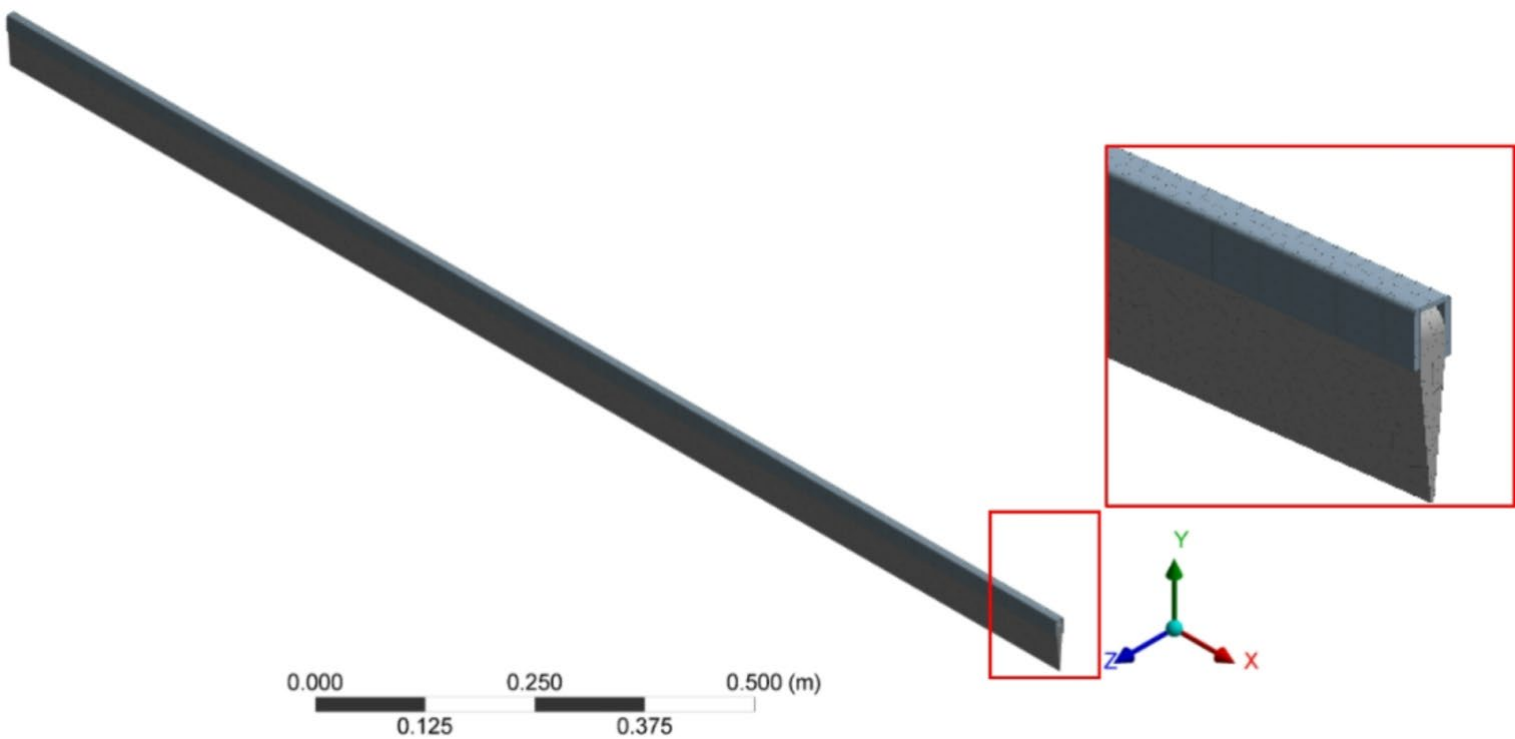
Mesh model of the wiper.

Figure 14 shows the mesh of the wiper used in this study, which was meshed into finite elements sized 5 mm × 5 mm with wall boundary conditions. The wiper model comprised 125,077 nodes and 57,328 elements, achieving an average element quality of 0.77, skewness quality of 0.27, and orthogonal quality of 0.82, indicating high meshing quality. The wiper material’s physical properties include a density of 1.10–1.25 g/cm³, hardness of 30–50 Shore A, and tensile strength of 4–12 MPa. It also exhibits high elongation (200–700%), low compression set (15–30%), and performs effectively in temperatures ranging from − 60 °C to + 200 °C.

**Fig. 15:**
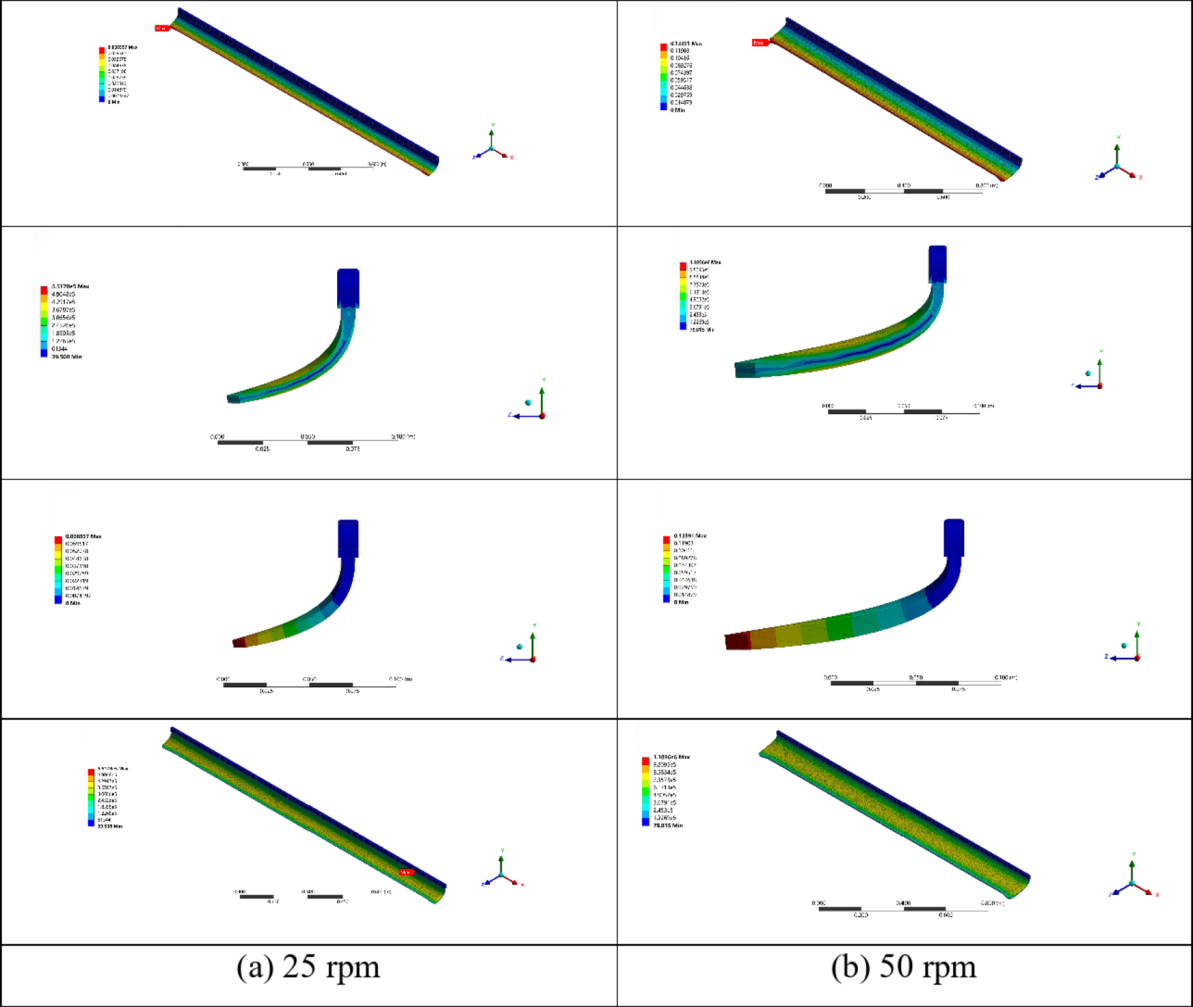
The wiper model shapes. (**a**) Motor speed 25 rpm, (**b**) Motor speed 50 rpm.

Figure 15 illustrates the structural analysis of wiper models at rotational speeds of 25 rpm and 50 rpm during the robot’s movement, highlighting the effects of speed on stress distribution and deformation. At 25 rpm, the wiper exhibits uniform deformation with lower stress concentrations, indicating stable performance at moderate speeds as shown in Fig. 15(a). In contrast, at 50 rpm, the wiper shows increased deformation and significant stress concentrations, particularly at the tip areas, due to higher friction forces with the greenhouse roof as shown in Fig. 15(b). This suggests that higher speeds could compromise the wiper’s structural integrity, potentially leading to material fatigue or failure over time. The comparison indicates that 25 rpm is more suitable for maintaining structural stability, while 50 rpm requires careful consideration of material strength and design reinforcement. The findings underscore the need to balance operational speed with durability requirements, suggesting that either speed control or enhanced material properties may be essential for optimizing wiper performance and ensuring effective cleaning operations in greenhouse environments.

### Structural analysis under wind load

**Fig. 16:**
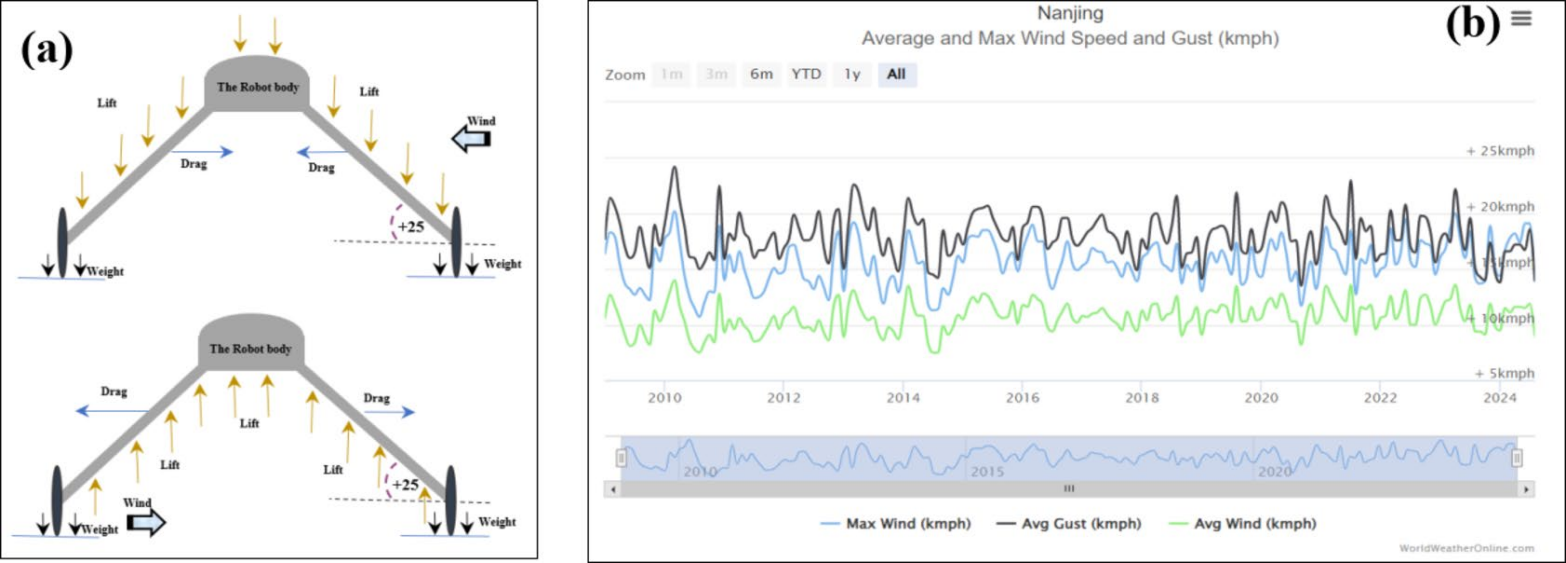
(**a**) vector diagram of forces, (**b**) wind speed data of Nanjing city (32°08′56″ N and 118°41′68″E) according to World Weather Online^40^.

The robot is subjected to two major forces: gravitational force, which acts downward on all structural elements due to their weight, and lift and drag forces, which act vertically and horizontally as shown in Fig. 16(a), resulting from the robot’s inclination angle 25°, causing the airflow to generate a significant upward lift force when wind passes over the robot body. Wind conditions at the experimental site in Nanjing City (coordinates: 32°08′56″ N and 118°41′68″E) are generally mild, as illustrated in Fig. 16(b), with the maximum wind speed recorded at 20 km/h and average speed wind 12.8 km/h under normal conditions. The interaction between the robot and greenhouse roof structure was analyzed using coupled FEA simulations. Results showed that the maximum deflection of the greenhouse roof under full robot loading was 12.3 mm, well within the acceptable safety limits of 15 mm. The stress distribution patterns confirmed that the robot’s weight distribution system effectively prevents any potential damage to the greenhouse panels, with peak stress values remaining 40% below the panels’ yield strength even under extreme operating conditions.

#### Wind speed impact on robot performance

**Fig. 17:**
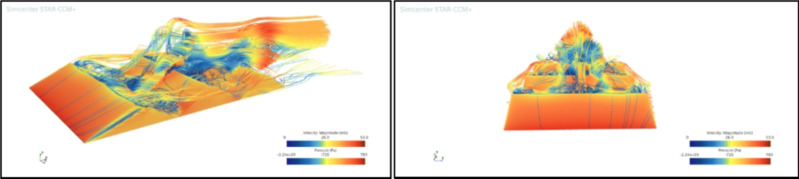
CFD simulation of velocity and pressure distribution.

Figure 17 shows the flow visualization results from the Simcenter STAR-CCM + simulation revealing complex flow characteristics around the geometry, with streamlines colored by velocity magnitude and pressure distribution. An analysis was conducted to evaluate the impact of wind speed on the robot by exposing it to conditions ten times greater than the average wind speed of Nanjing City, which is 12.8 km/h according to World Weather Online. The test was performed at a wind speed of 126 km/h. This increased wind speed was chosen to assess the robot’s efficiency and stability under extreme conditions, as the robot will be installed on top of a greenhouse where high wind speeds pose a significant risk. The analysis aimed to determine the robot’s vulnerability to potential falls and ensure its robustness in the face of abnormal wind fluctuations. The velocity scale ranges from 0 to 53.8 m/s, highlighting high-speed flow regions, particularly around curved or angled sections, while the pressure distribution ranges from approximately − 2240 Pa to 783 Pa, indicating areas of flow separation and recirculation where negative pressures dominate. These turbulent flow patterns suggest significant drag and energy losses, impacting system performance. High-velocity zones with low pressure can increase drag, while high-pressure regions near stagnation points indicate impingement effects. The velocity scale ranges from 0 to 53.8 m/s, highlighting high-speed flow regions, particularly around curved or angled sections, while the pressure distribution ranges from approximately − 2240 Pa to 783 Pa, indicating areas of flow separation and recirculation where negative pressures dominate. These turbulent flow patterns suggest significant drag and energy losses, impacting system performance. High-velocity zones with low pressure can increase drag, while high-pressure regions near stagnation points indicate impingement effects.

In conclusion, this analysis has provided valuable insights into how wind flow affects the design, demonstrating its efficiency and high performance even under extreme weather conditions, specifically at wind speeds ten times greater than typical normal conditions. This confirms the design’s robustness and effectiveness in challenging environments.

#### Pressure distribution analysis

**Fig. 18:**
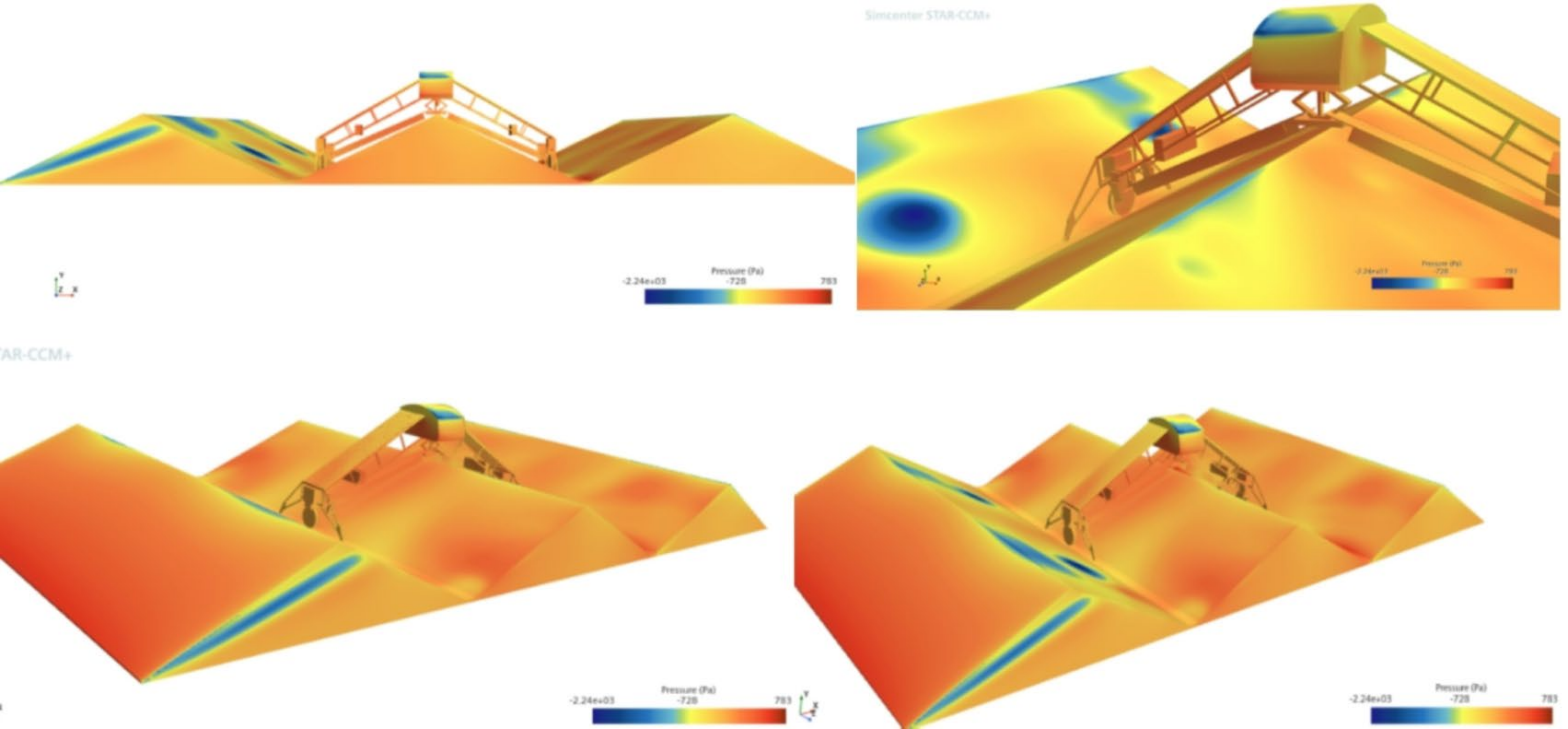
Pressure distribution on the surface from CFD simulation.

The pressure distribution analysis, conducted using Simcenter STAR-CCM+, reveals the interaction between the greenhouse roof cleaning robot and the surrounding environment as shown Fig. 18. The simulation demonstrates variations in pressure across the robot’s surface and the roof, highlighting areas of high and low pressure. Notably, regions with negative pressure (depicted in blue) indicate zones of potential aerodynamic lift or drag forces acting on the robot, which can influence its stability and operational efficiency. The pressure gradients around the robot’s cleaning arm and structural components show how air flow dynamics affect the cleaning performance, especially in maintaining close contact with the roof surface. Understanding these pressure variations was crucial for optimizing the robot’s design to enhance cleaning effectiveness while minimizing energy consumption and ensuring stable operation under varying environmental conditions.

## Conclusion

This study presents a comprehensive analysis of an innovative greenhouse roof cleaning robot, with a total operational weight of 142 kg including all components. The robot’s design incorporates several unique features: a frame structure optimized for standard greenhouse dimensions (4 m width, 40 m length, 3 m height), a 500 W solar power system with five flexible panels, and an integrated cleaning mechanism combining brushes, wipers, and water sprinklers.

Structural analysis demonstrated the robot’s durability under both static and dynamic conditions, with the distributed load of 0.42 kN/m² remaining well within the greenhouse roof’s design capacity of 0.96 kN/m². Adjustable brush/wiper heights significant improvement in light transmittance. CFD simulations validated the robot’s stability under extreme wind conditions of 126 km/h, ten times greater than normal operational conditions, with pressure distributions ranging from − 2240 Pa to 783 Pa.

**Table 4:** List of abbreviations.

Abbreviation	Full term	Abbreviation	Full term
*U*_*j*_	Velocity component in the j-direction (m/s)	CFD	Computational Fluid Dynamics
*ν*	Kinematic viscosity (m²/s)	ANSYS	simulation software
*ν*_*t*_	Turbulent viscosity (m²/s)	Simcenter STAR-CCM+	TSoftware for CFD and multiphysics
*P*_*k*_	Dimensionless (model constant)	k-ε	Turbulence model for predicting turbulent flow
*CRA*	Cement Removal Agent	PV	Photovoltaic.
*σ*_ε_	Dimensionless (constant)	DC	Direct Current
*C*_*1ε*_	Dimensionless (constant)	FEA	Finite Element Analysis
*C*_*2ε*_	Dimensionless (constant)	c_i_	Coefficient
*N*_*max*_	Maximum cleaning capacity (M/S)	L	Basis function (m)
*B*	Cleaning width (m)	θ	Theta (°).
*V*_*max*_	Maximum cleaning speed (m/s)	N	Dimensionless (no units)
*M*	Angle (°).	m	The object’s mass (kg)
*EI*	Flexural stiffness (N·m²).	MPPT	Maximum Power Point Tracking
l	Length (m).	Q_µN_	Shear force due to normal contact and frictional forces (N)
I	The line current strength (ampere)	Q_C_	shear force due to centrifugal forces (N)
Q	Shear force at bending (N)	V	The potential strength (volt)
P	The normal force of the cross-section (N)	dL	The differential brush length (m)
L1	The length of the hair at a certain point (m)	A	The cross-sectional area of the bristles (m)
ρ	The bristle density (kg/m^3^)	µN	The friction between the bristles and the plastic or glass
N	The normal force (N)	R	The distance between the bristle tip and the origin (mm)

Performance optimization studies revealed that a 25 rpm operating speed provides the ideal balance between cleaning efficiency and structural stability, compared to 50 rpm which induced higher stress concentrations and deformation. This research introduces a greenhouse cleaning robot powered by a 500 W solar system and equipped with an integrated brush-wiper-sprinkler system, enabling fully autonomous, lightweight, and wind-resistant operation for improved light transmittance and durability, representing a significant advancement over traditional and existing cleaning methods in terms of efficiency, sustainability, and adaptability. The robot’s performance is limited by its operational speed (0.35 m/s), battery life, reduced effectiveness on complex roofs, and maintenance requirements, while its utilization is constrained by adaptability to different greenhouse designs and initial cost, highlighting areas for future improvement.

Future developments will further focus on integrating AI-based navigation, real-time cleanliness monitoring, and optimized cleaning patterns to enhance operational efficiency and energy utilization.

Complete list of abbreviations used in this study can be found in Table 4.

## Data Availability

The datasets generated during and/or analyzed during the current study are available from the corresponding author upon reasonable request. In E-mail addresses: wangxiaochan@njau.edu.cn (X. Wang), aminahmed@m-eng.helwan.edu.eg (A. Amin).
